# Practical Anatomy

**Published:** 1867

**Authors:** 


*Practical Anatomy.*—Our readers have probably noticed in the secular press a strongly sensational account of the arrest of a couple of American citizens of African descent *in flagrante delictu*, having in possession several *cadavera* evidently designed for some anatomical theatre. It was supposed at first, from their proximity to the institution, that they were designed for the Rush College, but subsequent investigation showed, as was in fact the case, that in that iniquity, (if it be an iniquity,) Rush College, or any of its Faculty, had no part or lot. The morning dailies touched upon the affair merely as a matter of news, without any effort at a sensation, or to inflame the ignorant prejudices of the populace. But the evening sheets ascended to the highest peak of vituperative invective, and descended to the lowest depths of hoarse scurrility in their comments upon the matter. Everything was said that could be said to excite a mob spirit, but, fortunately, the Chicago public are too thoroughly accustomed to such diatribes to rise under the plentiful ferment, and in their great wrath smite things incontinently. As imaginative exercises are necessary to secure the sale of the evening prints, this course might be passed over without comment, were it not for the total recklessness of feeling manifested. The poor "resurrectionists," in their unpleasant and hazardous work, at least sought the cover of night and secrecy, and did everything in their power to prevent the feelings of any person from being lacerated. Theirs was a necessary work—it must be done by somebody, all admit so much; the offence if any was a Spartan one—*being discovered.* The "resurrectionist" would have scorned, even to have secured his own safety, to harrow up the feelings of friends by the slightest hint of the identity of the bodies seized. Not so the evening press. It paraded its anxiety for identification as an evidence of a peculiarly elevated type of Christian sympathy for the friends of the dead. It attacked venomously and vindictively, not only the poor scions of Africa, but some unknown party, whispered to be of higher station, as the worst of possible villains.

And when the representative of the JOURNAL, and in so far of the Medical Profession, stood in the Police Court, and risked his hardly-won estate upon the faith of the humble colored men who stood cowering in the prisoner's dock, facing the weeping friends, whose heart-wounds were torn open afresh by the incidents of the time—in his heart of hearts he placed the responsibility for all that suffering and all those tears, over which the reporters gloated in the evening press, not upon the poor tools whom Science, and Humanity in its widest meaning, had commanded to their obscure and dangerous work, but to that press which ransacked the nooks and corners of the town to find those over whose sorrows they could become sentimentally brutal, and thus sell their latest edition, illuminated with glaring capitals, and studded with thickly standing exclamation points.

The utter and shameless brutality of the act is its own damning commentary.

But whatever becomes of the present prisoners, the storm awakened by this occurrence will leave the atmosphere clearer. "Out of this nettle *danger*, the profession will pluck the flower *safety.*"

A calm, dispassionate, and very able writer in the *Tribune*

(whose article we wish our space would permit us to reproduce in full) discussed the subject in a manner, and with a force which the evening press was totally unable to meet. Never was rout more thorough—never was overthrow more annihilating.

This result, however, in itself is of little consequence—the great point gained is, that all parties admit the necessity of legislation, of which now there is a "plentiful lacking" in the State of Illinois, and in fact in most other Western States.

The secular press has in almost express terms guaranteed their support to *some* method, at least, of protection to both the living and the dead.

Let the Local Societies, the Colleges, the State Society, soon to meet, and whoever has personal or official influence with the Governor of the State, use that influence to have him name this as a subject requiring legislation at the extra session, if such a session is to be convened. If not, then bring every honorable appliance to bear on the next regular session.

Meanwhile, let it be remembered that there is now no law on the subject. Whatever bears upon it at all is but a vile opposition—an intolerance unworthy even of the dark ages which gave it birth.

The *Chicago Times*, looking at the thing practically, observes:

"It is both an absurdity and a tyranny, to hold the Physician accountable for a lack of knowledge, and, at the same time, to hold him accountable when he endeavors to supply himself with that knowledge. It is precisely the case of the Israelites, whom their Egyptian task-masters compelled to make bricks without straw.

"There are many bodies which can as well as not be used for the purposes of dissection. Every day there are men and women dying, at our public institutions, of charity, whom nobody knows, and who are buried at the public expense, and forgotten. We do not urge the giving of such bodies to medical classes *because* they are poor and friendless; but for the reason that they have no relatives to be shocked or horrified over this disposition of their remains.

"The merits of the whole question turn upon the feelings of surviving friends; and not upon any desecration attending a dissection. It is certainly as little a desecration to contribute to human knowledge at the dissecting table, as it is to become the prey of hideous worms, or to become swollen with gases, or blackened by decay. As between the two, the former is much the less shocking, barring the tender and political affection that clings to the grave which incloses a friend.

"It is not to be expected that people will willingly or unwillingly turn over the bodies of their friends for dissection; nevertheless, they should be willing to put the Physician in a position that will enable him to achieve that knowledge which is demanded of him. To do this, he should be provided with subjects for dissection; and until this shall be done, we may be certain, beyond all doubt, that the trade of the resurrectionist will be pursued with energy. Penalties will not prevent body-snatching. The only remedy, and the true one, is to make it unnecessary, by turning over for dissection the bodies of criminals, and of friendless paupers."

The bill rejected at the late session of the Illinois Legislature, receiving the votes of "scarcely a respectable minority," was as follows:

"*An Act to promote Medical Education.*

"It shall be lawful, in cities whose population exceeds twenty-five thousand (25,000) inhabitants, to deliver to the professors and teachers in medical colleges in this State, and for said professors and teachers to receive, for the purpose of medical study, the body of any deceased person, *provided*, that said body shall not have been claimed for interment by any relative or friend of the deceased, within twenty-four hours after death; *provided* also, that no one known to have relatives or friends, or detained for debt, or as a witness, or on suspicion of crime, or of any traveller, shall be so delivered, and *provided* also, that in case the body of any one so delivered or received shall be subsequently claimed by any relative or friend, it shall be given up to said relative or friend for interment, and it shall be the duty of the said professors and teachers, decently to bury in some public cemetery the remains of all bodies after they have answered the purposes of study aforesaid, and for any neglect of this provision of this act, the party so neglecting shall pay a fine of not less than twenty-five dollars ($25) nor more than fifty dollars ($50,) to be appropriated to the health department of said cities. The bodies delivered to professors and teachers, or received by said professors and teachers under this act, shall be used for the purposes of medical and surgical study alone, and in this State only, and whosoever shall use such body for any other purpose, or shall remove such body beyond the limits of this State, or in any manner traffic in the same, shall be deemed guilty of a misdemeanor, and shall, on conviction, be imprisoned for a term not exceeding one year, in a county jail.

"All laws, or parts of laws, inconsistent with this act, are hereby repealed.

"This act shall take effect from and after its passage."

This bill, essentially the same as the one now in force in the State of New York, or at least something like it, should be passed at the next session, even though there is no money in it for the lobby.

